# Supplementary material for: Structural basis of RNA-guided DNA integration by type I CRISPR-associated transposases
Source: bioRxiv. 2026 May 18:2026.05.18.725949. Preprint. [Version 1] doi: 10.64898/2026.05.18.725949 (PMC13228271; doi:10.64898/2026.05.18.725949)
Supplement: Supplement 1 [file media-1.pdf]

EXTENDED DATA TABLES AND FIGURES

Extended Data Table 1 Cryo-EM data collection, refinement and validation statistics

ID, EMDB and PDB codeS	Cryo-EM structure of the *Pse*Cascade-TniQ-TnsC complex (EMD-57765) (PDB-30GT)	Consensus cryo-EM volume of the *Pse*-Cascade-TniQ-TnsC complex (EMD-57751)	TnsC-focused cryo-EM volume of the *Pse*Cascade-TniQ-TnsC complex (EMD-57750)	Cryo-EM volume of the *Pse*Cascade-TniQ complex (EMD-57757)	Cryo-EM volume of the *Pse*Cascade complex (EMD-57758)
**Data collection and processing**					
Magnification	130,000	130,000	130,000	130,000	130,000
Voltage (kV)	300	300	300	300	300
Electron exposure ($e^-/Å^2$)	59.672	59.672	59.672	59.672	59.672
Defocus range (μm)	-1.0 to -2.4 (0.2 steps)	-1.0 to -2.4 (0.2 steps)	-1.0 to -2.4 (0.2 steps)	-1.0 to -2.4 (0.2 steps)	-1.0 to -2.4 (0.2 steps)
Pixel size (Å)	0.325	0.325	0.325	0.325	0.325
Symmetry imposed	C1	C1	C1	C1	C1
Initial particle images (no.)	745,080	745,080	745,080	745,080	745,080
Final particle images (no.)	76,400	76,400	76,400	50,074	132,574
Map resolution (Å)	3.26 (composite)	3.07	3.44	3.19	2.86
FSC threshold	0.143	0.143	0.143	0.143	0.143
Map resolution range (Å)	n/a	2.6-6.0	3.0-7.0	2.5-8.0	2.5-6.0
**Refinement**					
Initial model used (PDB code)	AlphaFold3, 7U5D	n/a	n/a	n/a	n/a
Model resolution (Å)	3.0	n/a	n/a	n/a	n/a
FSC threshold	0.143	n/a	n/a	n/a	n/a
Model resolution range (Å)	3.0-3.4	n/a	n/a	n/a	n/a
Map sharpening *B* factor ($Å^2$)	n/a	58.0	65.2	52.6	71.6
Model composition		n/a	n/a	n/a	n/a
Non-hydrogen atoms	51,759				
Protein residues	5,959				
Nucleotide residues	165				
Ligands	ATP: 7, MG: 7				
B factors ($Å^2$) min/max/mean		n/a	n/a	n/a	n/a
Protein	53.14/241.07/115.81				
Nucleotide	72.66/309.92/143.87				
Ligand	71.01/148.12/104.08				
R.m.s. deviations		n/a	n/a	n/a	n/a
Bond lengths (Å)	0.006(0)				
Bond angles (°)	0.564(0)				
Validation		n/a	n/a	n/a	n/a
MolProbity score	1.86				
Clashscore	8.16				
Poor rotamers (%)	2.47				
Ramachandran plot		n/a	n/a	n/a	n/a
Favored (%)	0.00				
Allowed (%)	2.60				
Disallowed (%)	97.40				

ID, EMDB and PDB codes	Cryo-EM structure of the *Pse*Cascade-TniQ-TnsC complex bound to *Pse*TnsB-hook motifs<br>(EMD-57739)<br>(PDB-30GB)	Consensus cryo-EM volume of the *Pse*Cascade-TniQ-TnsC complex bound to TnsB-hook motifs<br>(EMD-57738)	TnsC-focused cryo-EM volume of the *Pse*Cascade-TniQ-TnsC complex bound to TnsB-hook motifs<br>(EMD-57737)
**Data collection and processing**			
Magnification	130,000	130,000	130,000
Voltage (kV)	300	300	300
Electron exposure (e^- /Å^2)	60.947	60.947	60.947
Defocus range (μm)	-1.0 to -2.4 (0.2 steps)	-1.0 to -2.4 (0.2 steps)	-1.0 to -2.4 (0.2 steps)
Pixel size (Å)	0.325	0.325	0.325
Symmetry imposed	C1	C1	C1
Initial particle images (no.)	329,059	329,059	329,059
Final particle images (no.)	138,834	138,834	138,834
Map resolution (Å)	2.9 (composite)	2.84	2.96
FSC threshold	0.143	0.143	0.143
Map resolution range (Å)	n/a	2.5-5.0	2.5-5.0
**Refinement**			
Initial model used (PDB code)	30GT, AlphaFold3	n/a	n/a
Model resolution (Å)	2.8	n/a	n/a
FSC threshold	0.143	n/a	n/a
Model resolution range (Å)	2.8-3.2	n/a	n/a
Map sharpening *B* factor (Å^2)	n/a	72.7	83.7
Model composition<br>Non-hydrogen atoms<br>Protein residues<br>Nucleotide residues<br>Ligands	 52,521<br>6,022<br>175<br>ATP: 7, MG: 7	n/a	n/a
B factors (Å^2) min/max/mean<br>Protein<br>Nucleotide<br>Ligand	 56.32/220.70/112.76<br>76.75/313.01/147.48<br>68.42/103.32/83.71	n/a	n/a
R.m.s. deviations<br>Bond lengths (Å)<br>Bond angles (°)	 0.003(0)<br>0.570(0)	n/a	n/a
Validation<br>MolProbity score<br>Clashscore<br>Poor rotamers (%)	 1.70<br>7.32<br>1.83	n/a	n/a
Ramachandran plot<br>Favored (%)<br>Allowed (%)<br>Disallowed (%)	 0.00<br>2.47<br>97.53	n/a	n/a

ID, EMDB and PDB codes	Cryo-EM structure of the PseCascade-TniQ-TnsC-TnsAB holocomplex<br>(EMD-57736)<br>(PDB-30GA)	Consensus cryo-EM volume of the PseCascade-TniQ-TnsC-TnsAB holocomplex<br>(EMD-57731)	Cascade-TniQ-TnsC-focused cryo-EM volume of the PseCascade-TniQ-TnsC-TnsAB holocomplex<br>(EMD-57730)	TnsC-focused cryo-EM volume of the PseCascade-TniQ-TnsC-TnsAB holocomplex<br>(EMD-57729)	TnsAB-focused cryo-EM volume of the PseCascade-TniQ-TnsC-TnsAB holocomplex<br>(EMD-57728)
**Data collection and processing**					
Magnification	130,000	130,000	130,000	130,000	130,000
Voltage (kV)	300	300	300	300	300
Electron exposure (e^- /Å^2)	60.059 (grid1) / 65.580 (grid2)	60.059 (grid1) / 65.580 (grid2)	60.059 (grid1) / 65.580 (grid2)	60.059 (grid1) / 65.580 (grid2)	60.059 (grid1) / 65.580 (grid2)
Defocus range (μm)	-1.0 to -2.4 (0.2 steps)	-1.0 to -2.4 (0.2 steps)	-1.0 to -2.4 (0.2 steps)	-1.0 to -2.4 (0.2 steps)	-1.0 to -2.4 (0.2 steps)
Pixel size (Å)	0.325	0.325	0.325	0.325	0.325
Symmetry imposed	C1	C1	C1	C1	C1
Initial particle images (no.)	1,919,871	1,919,871	1,919,871	1,919,871	1,919,871
Final particle images (no.)	49,606	49,606	49,606	49,606	49,606
Map resolution (Å)	3.21 (composite)	3.10	3.09	3.30	3.36
FSC threshold	0.143	0.143	0.143	0.143	0.143
Map resolution range (Å)	n/a	2.5-8.5	2.5-6.5	2.5-6.5	2.5-6.5
**Refinement**					
Initial model used (PDB code)	30GB, 9T7L, AlphaFold3	n/a	n/a	n/a	n/a
Model resolution (Å)	2.8	n/a	n/a	n/a	n/a
FSC threshold	0.143	n/a	n/a	n/a	n/a
Model resolution range (Å)	2.1-3.5	n/a	n/a	n/a	n/a
Map sharpening *B* factor (Å^2)	n/a	43.1	35.9	50.8	52.3
Model composition<br>Non-hydrogen atoms<br>Protein residues<br>Nucleotide residues<br>Ligands	 74407<br>8274<br>348<br>ATP: 7, MG: 13	n/a	n/a	n/a	n/a
B factors (Å^2) min/max/mean<br>Protein<br>Nucleotide<br>Ligand	 30.00/270.24/109.72<br>20.00/323.94/148.85<br>68.21/155.99/89.75	n/a	n/a	n/a	n/a
R.m.s. deviations<br>Bond lengths (Å)<br>Bond angles (°)	 0.004(0)<br>0.617(0)	n/a	n/a	n/a	n/a
Validation<br>MolProbity score<br>Clashscore<br>Poor rotamers (%)	 1.87<br>8.51<br>2.19	n/a	n/a	n/a	n/a
Ramachandran plot<br>Favored (%)<br>Allowed (%)<br>Disallowed (%)	 0.00<br>2.83<br>97.17	n/a	n/a	n/a	n/a

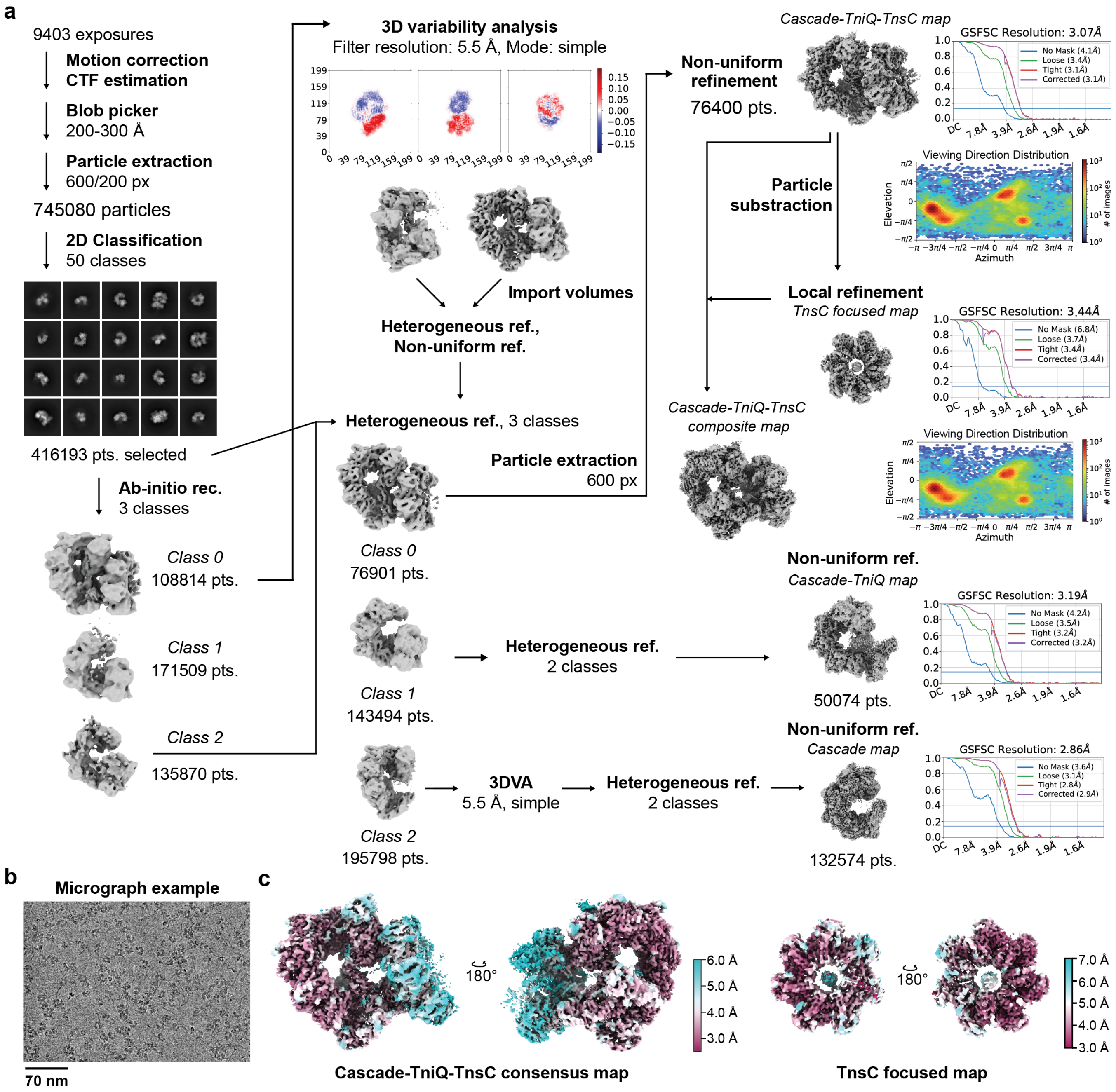

Extended Data Fig. 1. Cryo-EM data processing of the *Pse*Cascade-TniQ-TnsC targeting complex. a, Cryo-EM image processing workflow for the *Pse*Cascade-TniQ-TnsC complex. Resolution is determined by Fourier Shell Correlation (FSC) calculated from two independently refined half-maps. The gold-standard cutoff (FSC = 0.143) is marked with a blue line. Final maps were obtained for *Pse*Cascade-TniQ-TnsC (top, 3.07 Å), *Pse*Cascade-TniQ (middle, 3.19 Å), and *Pse*Cascade (bottom, 2.86 Å). **b**, Representative micrograph. **c**, Final consensus and locally refined electron density maps for *Pse*Cascade-TniQ-TnsC and TnsC only, coloured according to local resolution.

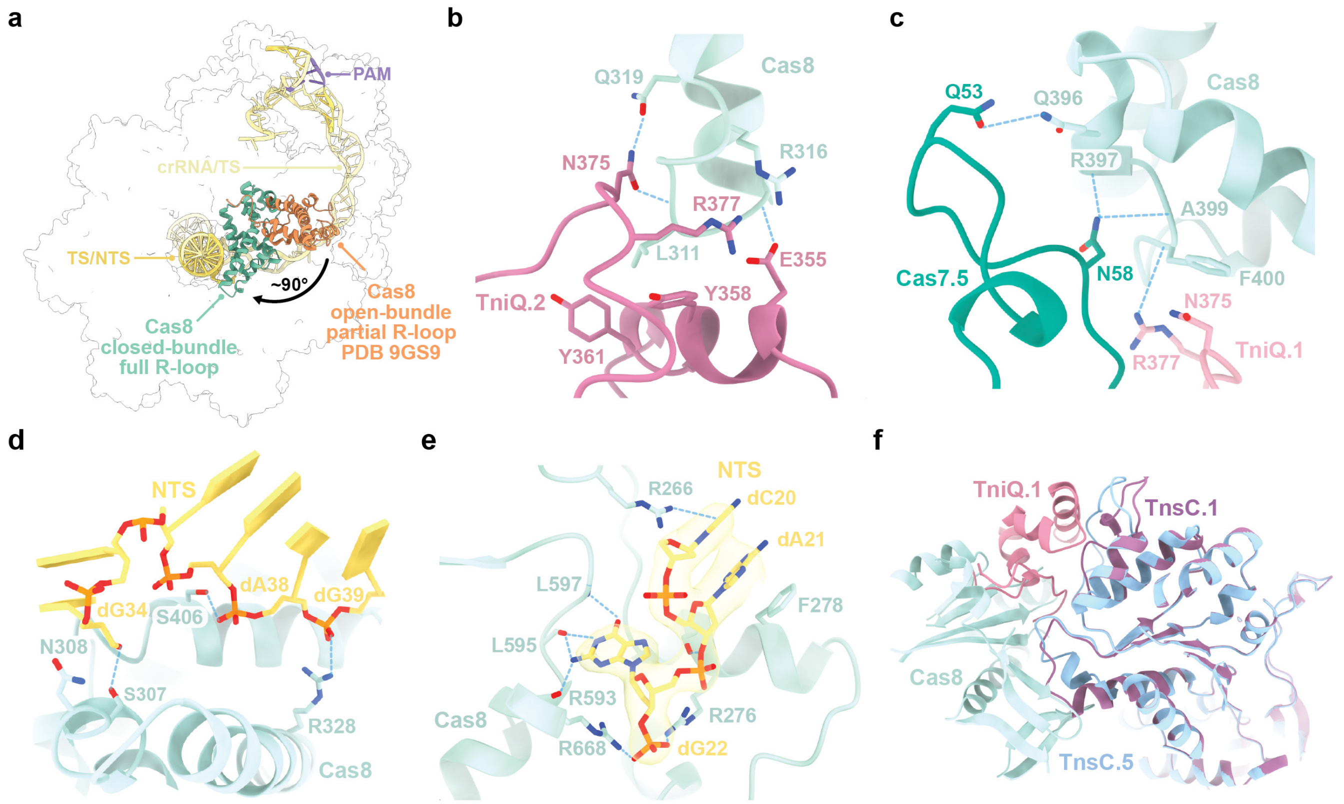

Extended Data Fig. 2. Domain rearrangements and interaction interfaces of Cas8. a, Rotation of the Cas8 bundle in the *Pse*Cascade complex with respect to its partial R-loop state (PDB 9GS9)[9]. **b-d**, Structural details of the interactions of the Cas8 helical bundle with TniQ.2 (**b**), TniQ.1 and Cas7.5 (**c**), and the PAM-distal non-target strand (NTS) (**d**). **e**, Binding of the displaced NTS by the Cas8 bundle. Bases are shown in stick representation, with corresponding cryo-EM density (transparent surface in yellow) superimposed . **f**, Superposition of TnsC.1-TniQ and TnsC.5-Cas8 interfaces.

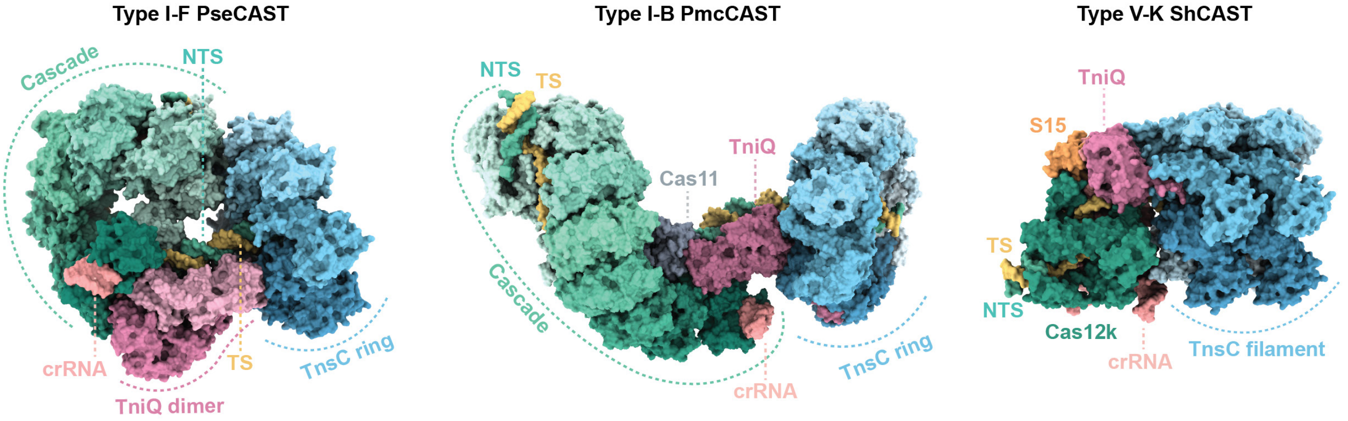

Extended Data Fig. 3. Structural comparisons of targeting complexes from representative CAST systems. Side-by-side views of type I-F *Pse*Cascade-TniQ-TnsC (this study), type I-B *Pmc*Cascade-TniQ-TnsC (PDB 8FF4)[41], and type V-K *Sh*Cas12k-S15-TniQ-TnsC (PDB 8EA3)[6]; components beyond the TnsC filament were removed for clarity). Analogous components are highlighted in the same colours across systems, and structures are aligned using the matchmaker function in ChimeraX.

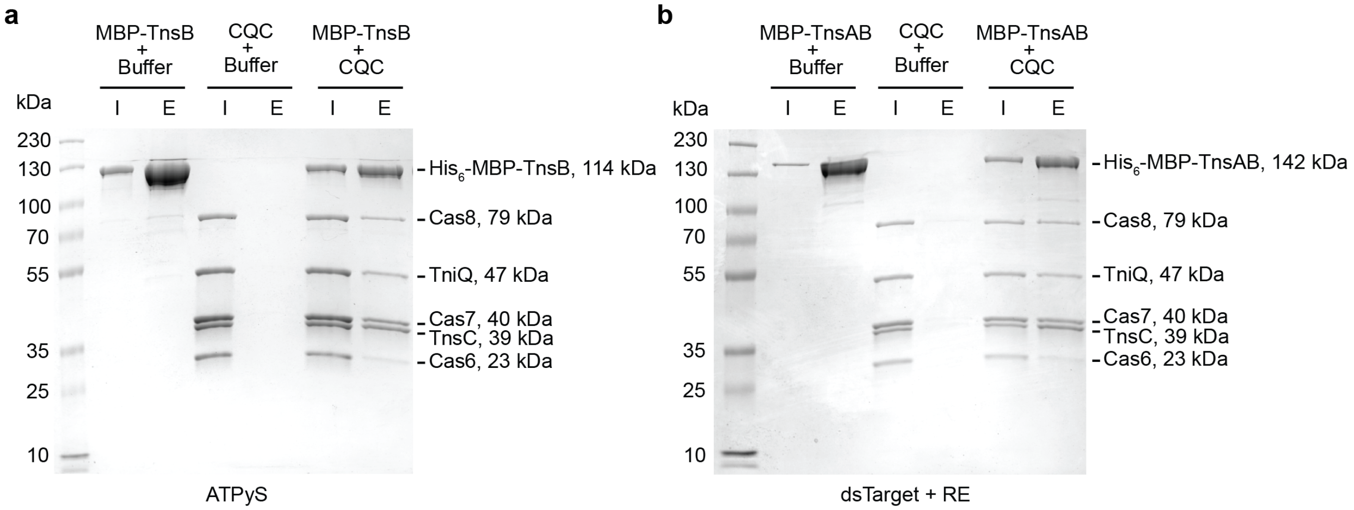

Extended Data Fig. 4. Co-precipitation of *Pse*Cascade-TniQ-TnsC by TnsB or TnsAB. a, Co-precipitation of Cascade-TniQ-TnsC by full-length His_6-MBP-TnsB in the presence of ATPγS and double-stranded target DNA (dsTarget). **b**, Reconstitution of the *Pse*Cascade-TniQ-TnsC-TnsB-hook complex for cryo-EM analysis. The complex was assembled using co-precipitation of dsTarget DNA-bound Cascade-TnsC-TniQ by amylose-immobilized full-length TnsAB bound to double stranded transposon right end (RE) DNA in the presence of ATP. CQC, Cascade-TniQ-TnsC; I, 5% input control; E, elution.

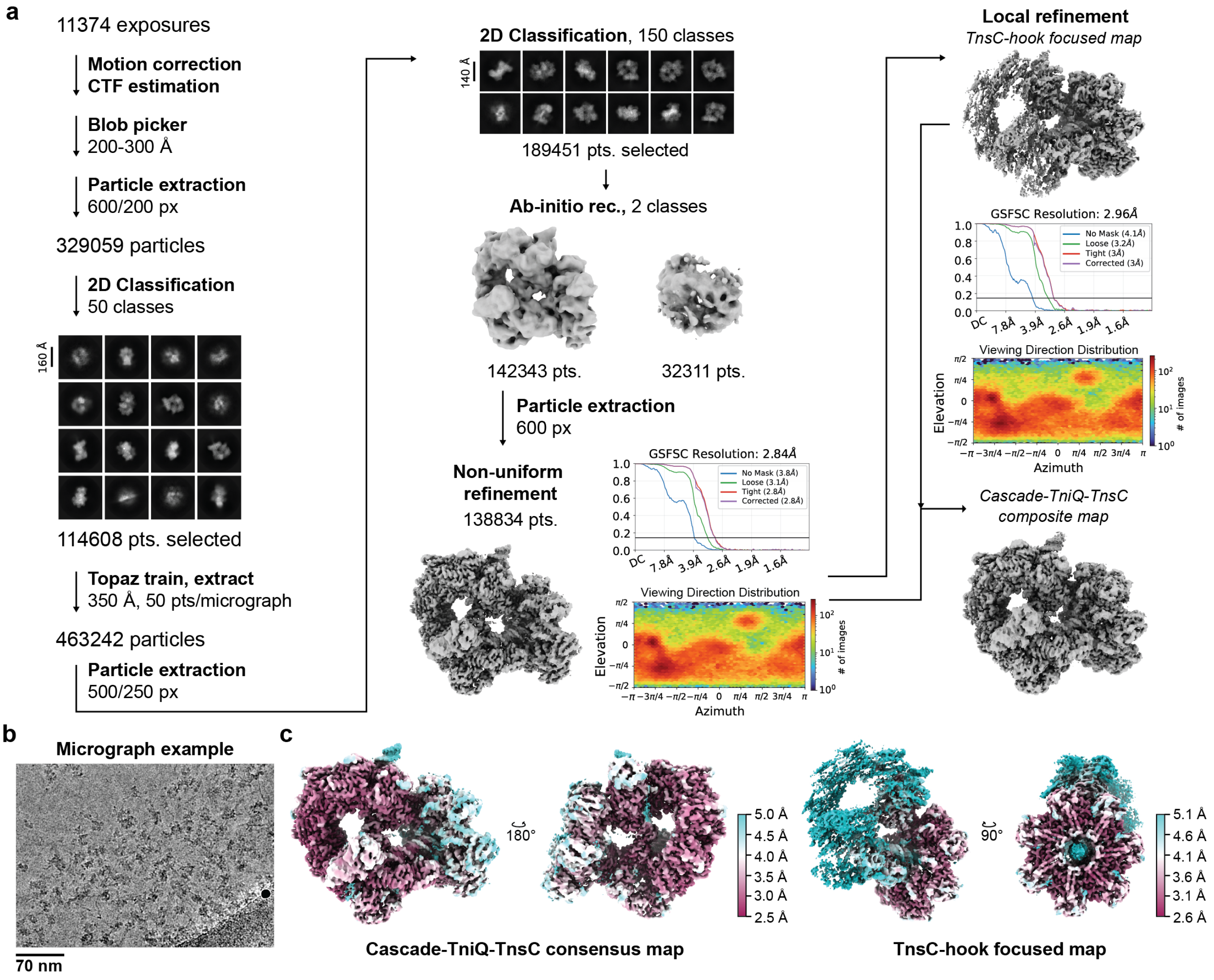

Extended Data Fig. 5. Cryo-EM data processing of the *Pse*Cascade-TniQ-TnsC-TnsB-transposase recruitment complex. a, Cryo-EM image processing workflow for the *Pse*Cascade-TniQ-TnsC-TnsB-hook complex. Resolution is determined by Fourier Shell Correlation (FSC) calculated from two independently refined half-maps. The gold-standard cutoff (FSC = 0.143) is marked with a black line. **b**, Representative micrograph. **c**, Final consensus and locally refined electron density maps for *Pse*Cascade-TniQ-TnsC-TnsB-hook and TnsC-hook only, coloured according to local resolution.

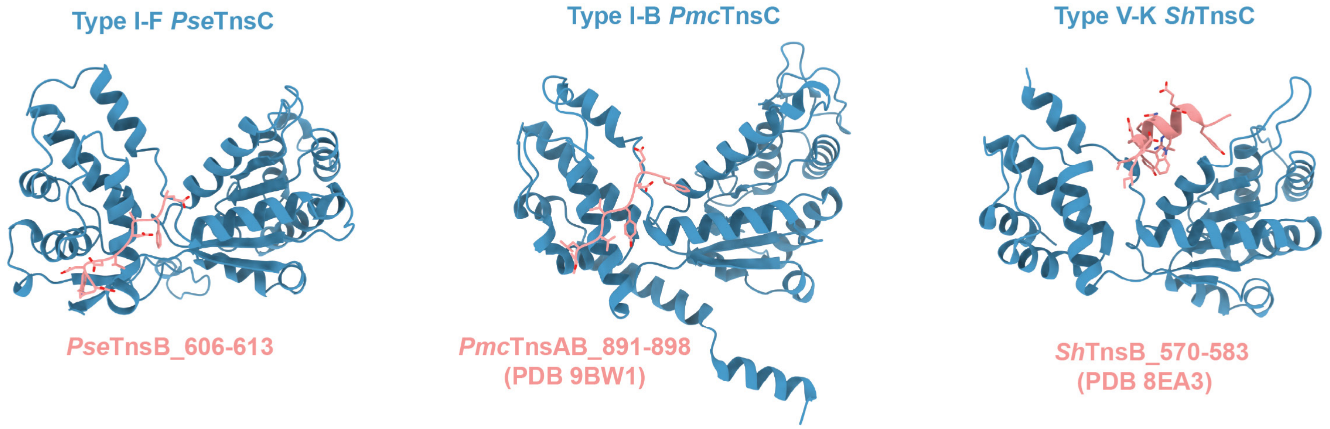

Data Fig. 6. Comparison of TnsB hook-bound TnsC structures. Side-by-side views of the TnsC-TnsB hook interfaces in type I-F *Pse*CAST (this study), type I-B *Pmc*CAST (PDB 9BW1), and type V-K *Sh*CAST (PDB 8EA3), shown in the same orientation.

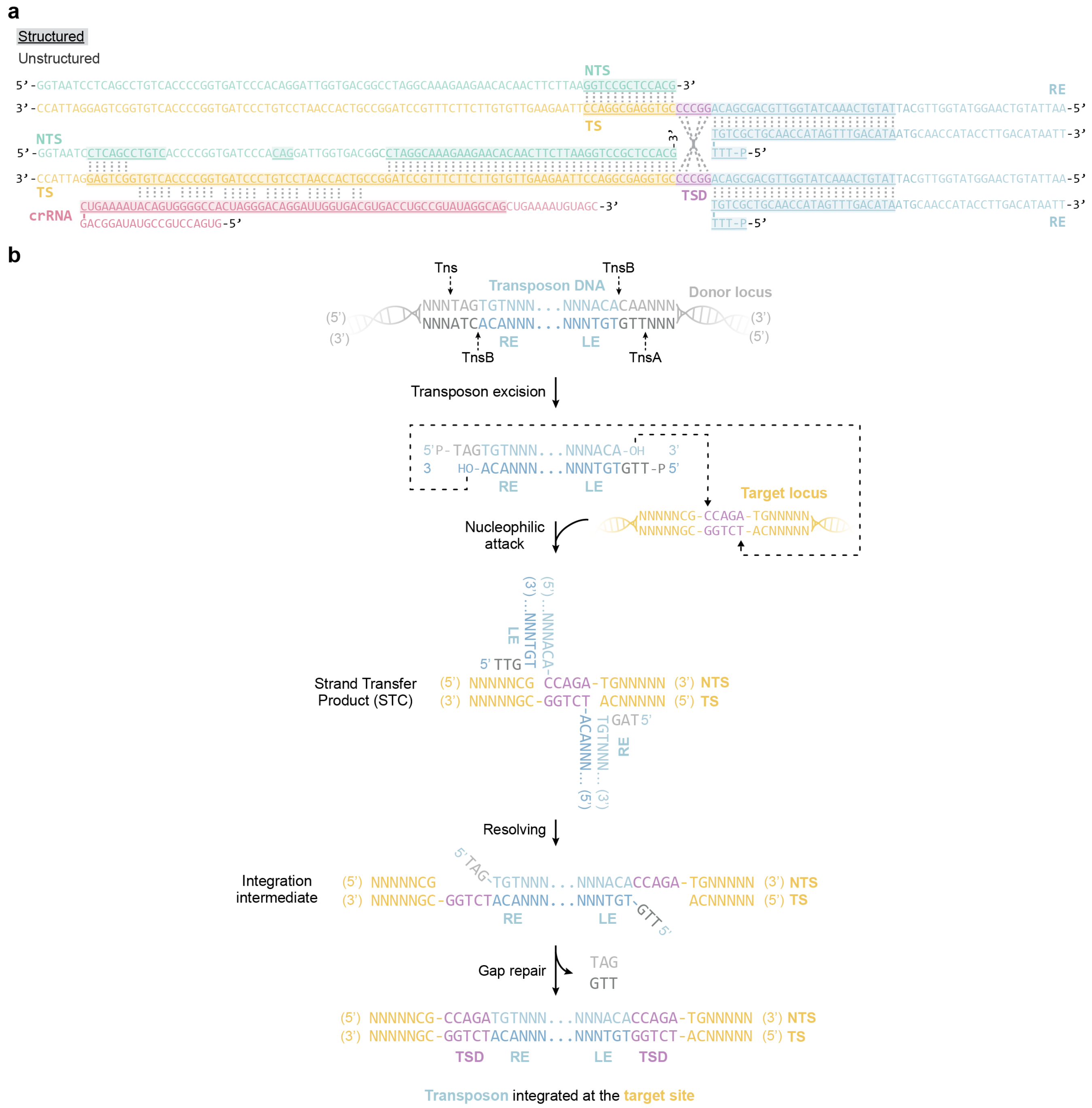

Extended Data Fig. 7. Transposition DNA constructs. a, Full-length sequence and base pairing of the oligonucleotides used for *Pse*CAST holocomplex reconstitution, coloured as in **Fig. 3a**. The structured portion modelled in the structure is highlighted. **b**, Sequence-level schematic diagram of the donor and target DNAs and the cut-and-paste transposition cycle of *Pse*CAST. The transposases TnsA and TnsB recognize and process the left (LE) and right (RE) ends, excising the transposon DNA, which is subsequently integrated at the target site through a strand-transfer intermediate by TnsB. TS: target strand; NTS: non-target strand; TSD: target site duplication.

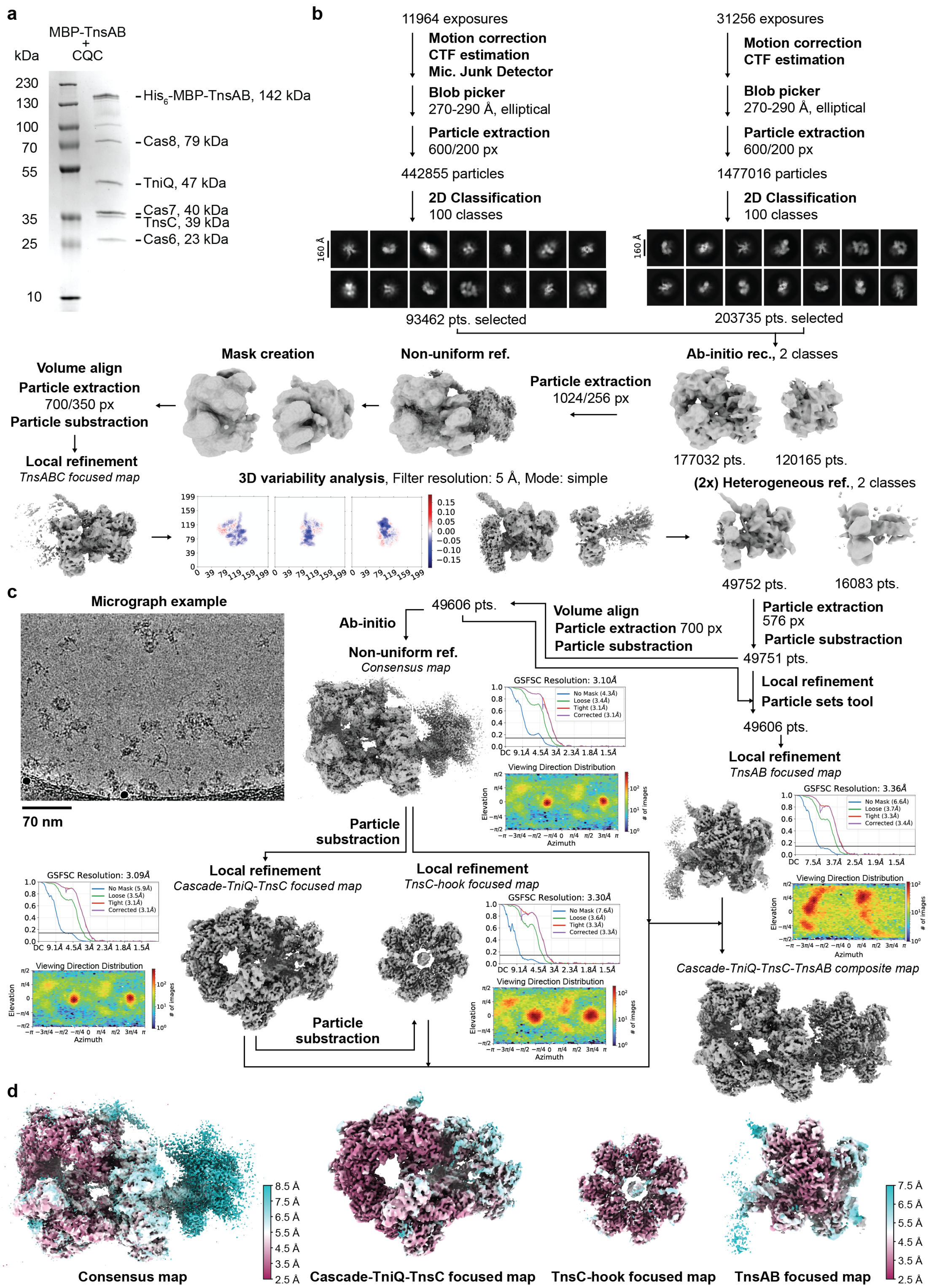

Extended Data Fig. 8. Cryo-EM sample preparation and data processing of the *Pse*Cascade-TniQ-TnsC-TnsAB holocomplex. a, SDS-PAGE of the eluate from the co-precipitation of *Pse*Cascade-TnsC-TniQ by amylose-immobilized full-length His_6-MBP-tagged *Pse*TnsAB bound to a strand-transfer DNA construct containing a target site, a pseudo-palindromic TSD and a double-stranded RE. The sample was used for cryo-EM analysis to obtain the *Pse*Cascade-TniQ-TnsC-TnsAB holocomplex structure. **b**, Cryo-EM image processing workflow for the *Pse*Cascade-TniQ-TnsC-TnsAB holocomplex. Resolution is determined by Fourier Shell Correlation (FSC) calculated from two independently refined half-maps. The gold-standard cutoff (FSC = 0.143) is marked with a black line. **c**, Representative micrograph. **d**, Global consensus electron density map of the *Pse*Cascade-TniQ-TnsC-TnsAB complex and locally refined volumes of the *Pse*Cascade-TniQ-TnsC, TnsC-hook, and TnsAB subcomplexes, coloured according to local resolution.
